# Advantages of Ta-Doped Sb_3_Te_1_ Materials for Phase Change Memory Applications

**DOI:** 10.3390/nano13040633

**Published:** 2023-02-05

**Authors:** Mingyue Shao, Yang Qiao, Yuan Xue, Sannian Song, Zhitang Song, Xiaodan Li

**Affiliations:** 1 State Key Laboratory of Functional Materials for Informatics, Shanghai Institute of Microsystem and Information Technology, Chinese Academy of Sciences, Shanghai 200050, China; 2 Center of Materials Science and Optoelectronics Engineering, University of Chinese Academy of Sciences, Beijing 100049, China; 3 The Microelectronic Research & Development Center, Shanghai University, Shanghai 200444, China

**Keywords:** high speed, thermal stability, crystallization, PCM

## Abstract

Phase change memory (PCM), a typical representative of new storage technologies, offers significant advantages in terms of capacity and endurance. However, among the research on phase change materials, thermal stability and switching speed performance have always been the direction where breakthroughs are needed. In this research, as a high-speed and good thermal stability material, Ta was proposed to be doped in Sb_3_Te_1_ alloy to improve the phase transition performance and electrical properties. The characterization shows that Ta-doped Sb_3_Te_1_ can crystallize at temperatures up to 232 °C and devices can operate at speeds of 6 ns and 8 × 10^4^ operation cycles. The reduction of grain size and the density change rate (3.39%) show excellent performances, which are both smaller than that of Ge_2_Sb_2_Te_5_ (GST) and Sb_3_Te_1_. These properties conclusively demonstrate that Ta incorporation of Sb_3_Te_1_ alloy is a material with better thermal stability and faster crystallization rates for PCM applications.

## 1. Introduction

Since the 1950s, the rapid development of non-volatile memories has greatly contributed to faster data exchange in the age of the Internet. Phase change memory has attracted attention for its fast programming speed, excellent re-writable characteristics, good stability, and logical compatibility.

Breitwisch [1] showed that there is no clear physical limit to PCM so far, and that device can still phase change when the thickness of the phase change material is reduced to 2 nm. Therefore, PCM is considered to be the most likely solution to the storage technology problem and then to replace the current mainstream storage products as one of the new generations of non-volatile semiconductor memory devices that will be common in the future.

Raoux illustrates that phase change materials based on sulphur compounds have a transition between high and low resistance values when excited by an electric field. Moreover, sulphur-based materials can achieve fast nanosecond transitions between amorphous and crystalline states when thermally induced by a pulsed laser. The structural differences between these two material forms lead to a significant difference in their macroscopic optical reflectivity, and this difference enables the storage of two stable data states [2].

As a classical phase change material, Ge_2_Sb_2_Te_5_ (GST) has the potential to be improved in terms of speed and stability when used in practical applications [3,4]. Since the Sb-Te system is a growth-dominated phase change material [5], the growth-dominated crystallization behaviour leads to a faster crystallization rate [6,7,8]. PCM devices based on Sb_3_Te_1_ can achieve higher operation speed on the premise of improving data retention capability compared with GST. Based on Ghosh’s work [9], Stefan proposed a new version of the Sb-Te phase diagram, in which only the γ phase is used to determine all phase compositions between pure Sb and Sb_2_Te_3_, with the atomic percentage of Te in the phase ranges from 11.4 at.% to 56.9 at.% [10]. According to the study, it can be proved that the Te content in the Sb_3_Te_1_ is 25 at.%, which is a single phase.

In this study, the microstructural characterization and electrical properties of the Ta-doped Sb_3_Te_1_ films and devices were investigated. Ta_1_._45_Sb_3_Te_1_ has enhanced thermal stability, reduced grain size, and increased the switching speed of PCM devices. The improved performance of Ta-doped Sb_3_Te_1_ materials is analyzed in the paper through changes in the microstructure of the films.

## 2. Experiment

By using a Ta target and a Sb_3_Te_1_ target during magnetron co-sputtering, Sb_3_Te_1_ and Ta_x_Sb_3_Te_1_ (Ta_x_ST31) films were produced on SiO_2_ substrates. The sputtering power controls the composition of the designed film. Production of pure Sb_3_Te_1_ films from Sb_3_Te_1_ targets are sputtered onto SiO_2_ substrates using a 20 W RF magnetron. The RF magnetron power of the Ta target was set to 6 W and 8 W while the Sb_3_Te_1_ target was co-sputtered at 20 W to deposit two different ratios of Ta_x_ST31 films on the SiO_2_ substrate. The ratios of the Ta_x_ST31 films were experimentally evaluated using energy dispersive spectroscopy (EDS) and recorded as Ta_1_._08_Sb_3_Te_1_ (TaST31-1), Ta_1_._45_Sb_3_Te_1_ (TaST31-2).

The experiment was then carried out with prepared 60 nm films of pure Sb_3_Te_1_, TaST31-1, and TaST31-2. The R-T measurements were performed at a 20 °C/min heating rate. The films were heated at a rate of 20 °C/min while under vacuum, and the crystallization temperature of the films was calculated for comparison and analysis.

Furthermore, the microstructure of 150 nm pure Sb_3_Te_1_, TaST31-1, and TaST31-2 films were explored using X-ray diffraction (XRD) for lattice information of the films. Furthermore, 30 nm thick films of pure Sb_3_Te_1_ and TaST31-2 were deposited on the Cu grid, and the transformation process was also studied by transmission electron microscopy (TEM) to investigate the structural evolution. Additionally to this, the 40 nm TaST31-1 and TaST31-2 films were characterized using X-ray reflectivity (XRR) to observe the density variation of the films. The 150 nm pure Sb_3_Te_1_ and TaST31-2 films were selected for analysis of their chemical bonding properties by applying X-ray photoelectron spectroscopy (XPS).

Finally, “T-shaped” PCM devices were fabricated to test their electrical properties. Pre-industrial delivery was carried out using 0.13 µm complementary metal-oxide semiconductor technology. A sputtering method was used to deposit a 100 nm thick phase change material and a 30 nm thick titanium nitride as an adhesion layer on a 190 nm diameter tungsten heated electrode. Eventually, resistance–voltage (R–V) measurements and endurance tests were carried out utilizing a Tektronix AWG5002B pulse generator and a Keithley 2400 C source meter.

## 3. Results and discussion

### 3.1. Effect of Ta Doping on Crystalline Properties and Microstructure

In this research, resistance–temperature (R-T) test experiments were first carried out on 60 nm films of pure Sb_3_Te_1_, TaST31-1, and TaST31-2.

**Figure 1:**
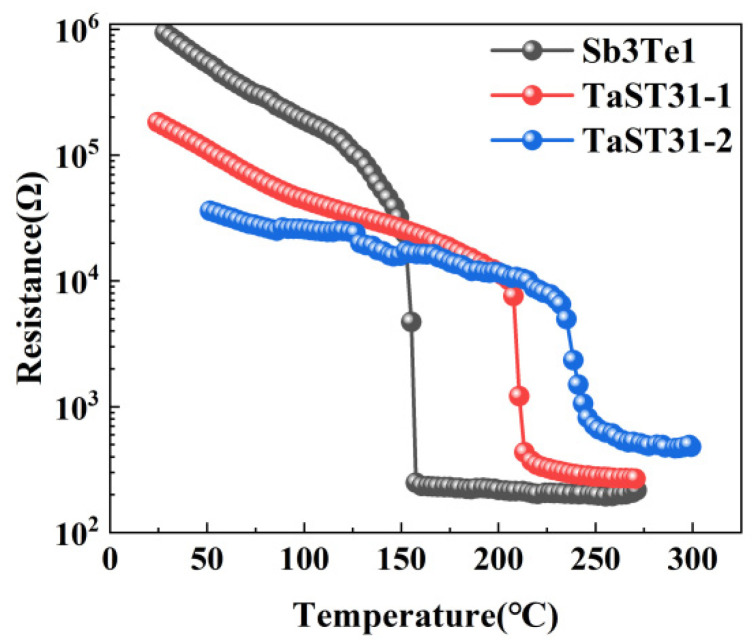
The R–T measurement results for Sb_3_Te_1_, TaST31-1, and TaST31-2.

Figure 1 shows the curve of resistance as a function of temperature for the three different components of the films. The resistivity of the films first reduces gradually with rising temperature. Before the crystallization temperature is attained, resistance rapidly decreases. The minimum value of the first-order inverse of the R-T curve is used to compute the crystallization temperature (Tc). The crystallization temperatures of pure Sb_3_Te_1_, TaST31-1, and TaST31-2 are 152 °C, 203 °C, and 230 °C, respectively. The crystallization temperature of the Sb_3_Te_1_ film can be increased by Ta doping. The substance GST crystallizes at a temperature of about 150 °C, according to studies [11,12]. In comparison, it can be found that the addition of the material Ta can increase the crystallization temperature by 50 °C to 80 °C, which greatly improves the thermal stability of the material.

**Figure 2:**
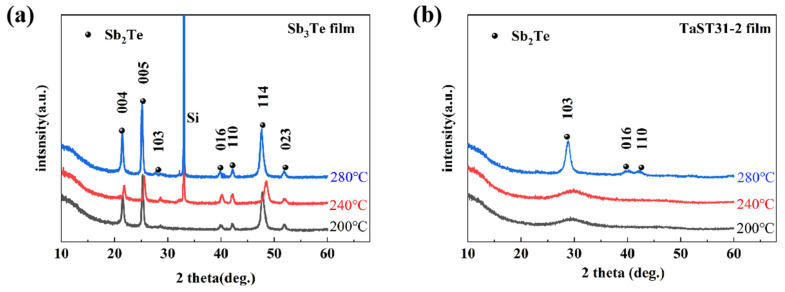
XRD curves of (**a**) Sb_3_Te_1_ and (**b**) TaST21-2 films at different temperatures.

The lattice information of the Sb_3_Te_1_ and TaST31-2 films were characterized by XRD measurements, as shown in Figure 2a,b. The Sb_3_Te_1_ and TaST21-2 films with a thickness of 150 nm were annealed in N_2_ at 200 °C, 240 °C, and 280 °C for 5 min. The diffraction peaks of Sb_3_Te_1_ film appeared at 200 °C, which is corresponding to the hexagonal Sb_2_Te phase. As observed in Figure 2b, the crystallization of TaST31-2 film is delayed, and the diffraction peak appeared at 240 °C. By comparing the crystal orientation indexes in Figure 2a,b, the TaST31-2 film shows an increase in the intensity of the 103 peak and a decrease in the intensity of the 004 and 005 peaks compared to the pure Sb_3_Te_1_ films, while the 114 and 023 peaks no longer appear. It can clearly be observed that the diffraction peaks of the Ta-doped Sb_3_Te_1_ film broaden, but there are no diffraction peaks of the metal Ta or Ta-containing metal compounds, and no isolated Sb [13]. The results of the XRD curves indicate that the main crystal structure of the Ta-doped Sb_3_Te_1_ film has not changed. Moreover, the half-peak width of the Ta-doped Sb_3_Te_1_ film has become larger, which means that the grains have been refined.

**Figure 3:**
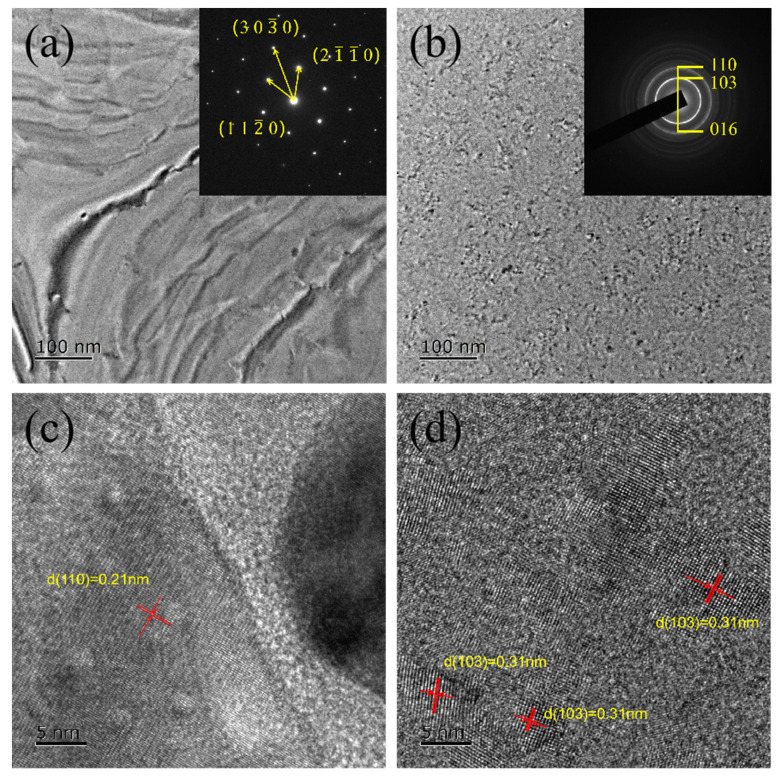
The BF TEM images and their corresponding SAED patterns of (**a**) Sb_3_Te_1_, (**b**) TaST31-2 films. The HRTEM images for (**c**) Sb_3_Te_1_ and (**d**) TaST31-2.

Transmission electron microscopy (TEM) enables a more visual observation of the grain size and crystalline phase. The test samples for TEM are 30 nm thick films of Sb_3_Te_1_ and TaST31-2 deposited on copper grids, which can be directly used for TEM observation and analysis. All samples were annealed in N_2_ at 280 °C for 5 min. BF TEM images of Sb_3_Te_1_ and TaST31-2 films and their associated SAED patterns are shown in Figure 3a,b, respectively. Figure 3c,d are HRTEM images of Sb_3_Te_1_ and TaST31-2 films, respectively. The comparison of BF images shows that the grain size of TaST31-2 film is significantly smaller than that of the Sb_3_Te_1_ film. It proves that after Ta doping, the crystallization behaviour of Sb_3_Te_1_ switches from being growth-dominated to nucleation-dominated. Meanwhile, the SAED pattern of the Sb_3_Te_1_ film shows single crystal diffraction spots, indicating that it has grown into large grains. In contrast, the intensity of diffraction rings that belong to 110, 103, and 016 crystallographic planes of hexagonal structure of Sb_2_Te emerged in the corresponding SAED pattern of TaST31-2 film and showed a small degree of discontinuity, further explaining the inhibition effect of Ta on grains [14]. It also can be found that the grains become uniform on the HRTEM images of TaST31-2 films. Sb_3_Te_1_ films have a grain size of at least 100 nm, which is reduced to 5–10 nm after Ta doping. Therefore, it is concluded that the grain size decreases and no other phases in Ta doping Sb_3_Te_1_ film. The small size of the grain contributes to reducing the thermal conductivity and resistance drift as well as the formation of a homogeneous phase, which helps to improve the heating efficiency and the reliability of the device [15].

**Figure 4:**
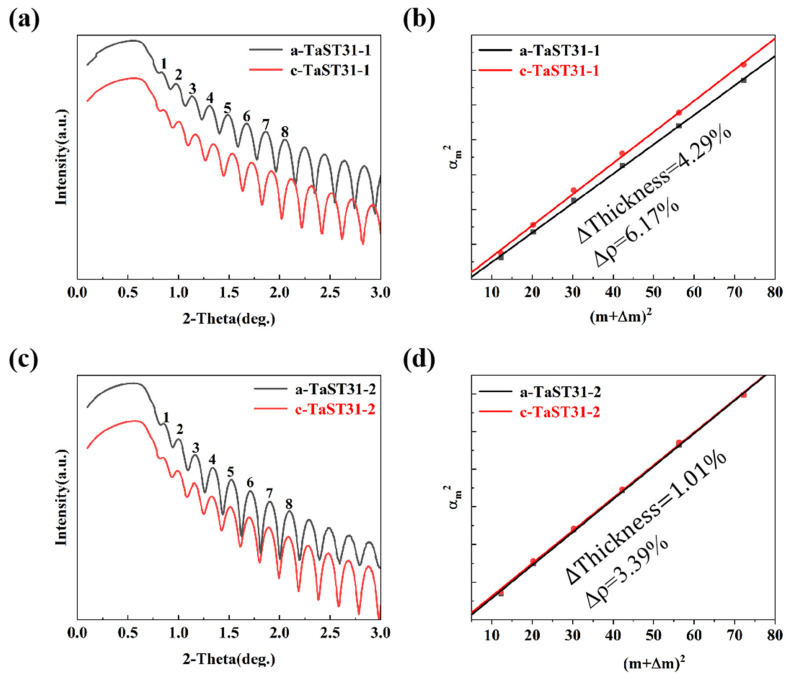
The density changes of the TaST31 films after crystallization. XRR curves of amorphous and crystalline (**a**) TaST31-1 and (**c**) TaST31-2 films. Bragg fitting curves of amorphous and crystalline films of (**b**) TaST31-1 and (**d**) TaST31-2 films.

Bruker D8 Discover’s X-ray reflectivity (XRR) investigations can be used to look at how the thickness and density of the films change both before and after crystallization. Figure 4a,c show the XRR diffraction images of the TaST31-1 and TaST31-2 films, respectively. The density change values for the amorphous and crystalline phases of the TaST31-1 and TaST31-2 films are shown in Figure 4b,d, respectively. The principle of operation of XRR is to take advantage of the reflection and refraction phenomena that occur on the surface of the sample when X-rays are reflected. The refracted light enters the interior of the sample and is reflected and refracted again at the Si substrate interface. The two reflected beams interfere to produce interference fringes [16]. Figure 4a,c show that the XRR curves of the Ta-doped Sb_3_Te_1_ films are wavy, with multiple peaks and troughs. The numbers 1–8 in the figures are the number of wave peaks. Based on the displacement of the peaks, fitting calculations are performed to show the thickness and density change corresponding to the amorphous to crystalline transition from Figure 4b,d. In the figures, the exact film thickness *d* can be deduced from the slope of the curve [${\left(\frac{\lambda }{2d}\right)}^{2}$] and the critical angle calculated from the intercept ($\alpha_{m}^{2}$) on the *y*-axis. The density change of the film is calculated from Equation (1):

(1) $$\nabla \rho =\frac{\rho_{mc}-\rho_{ma}}{\rho_{mc}}=\frac{\alpha_{\mathrm{cc}}^{2}-\alpha_{\mathrm{ca}}^{2}}{\alpha_{\mathrm{cc}}^{2}},$$

where $\rho_{mc}$, $\rho_{ma}$ are the density of the crystalline and amorphous TaST31, respectively, and $\alpha_{\mathrm{ca}}^{2}$, $\alpha_{\mathrm{cc}}^{2}$ are the critical angles of the XRR curve for the amorphous and crystalline TaST31. It was calculated that the rate of change in thickness for the TaST31-1 film is 4.29%, compared to 1.01% for the TaST31-2 film. Meanwhile, the rate of change in density was 6.17% for film TaST31-1 and 3.39% for film TaST31-2. The density change of the film TaST31-2 (3.39%) is lower than that of GST (6.8%). From this result, it can be concluded that the change in density decreases as the Ta percentage increases. This value of density change improves the durability of the material [17,18].

**Figure 5:**
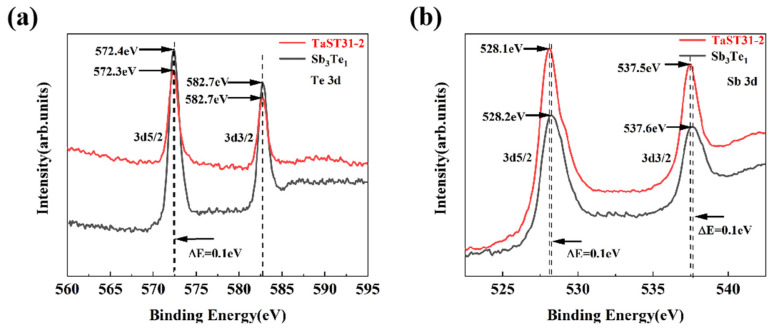
Sb_3_Te_1_ and TaST31-2 films’ XPS spectra after 260 °C annealing (**a**) Te 3d and (**b**) Sb 3d.

The chemical bonding properties of Sb_3_Te_1_ and TaST31 films in the crystalline form were examined by the XPS method in order to examine the influence of Ta doping. Figure 5a,b show the XPS spectra of Te3d and Sb3d, respectively. In Figure 5a, it can be seen that the bond energies of 572.4 eV and 582.7 eV in the Sb_3_Te_1_ film correspond to Te 3d5/2 and 3d3/2, respectively, and the bond energies of 572.3 eV and 582.7 eV in the TaST31-2 film correspond to Te 3d5/2 and 3d3/2, respectively. The result indicates that by doping Sb_3_Te_1_ with Ta element, the binding energy of Te will shift towards a lower binding energy. As shown in Figure 5b, it is also observed that the bond energy of Sb 3d5/2 is reduced from 528.2 eV to 528.1 eV and the bond energy of Sb 3d3/2 is reduced from 537.6 eV to 537.5 eV with lower binding energy. The electronegativities are 1.4, 2.05, and 2.1 for Ta, Sb, and Te elements, respectively [19], making a stronger Ta-Te bond than an Sb-Te bond. Ta tends to combine with Te to form more Ta-Te bonds and extra Sb-Sb bonds. The bond energy of the Ta-Te bond is larger than that of the Sb-Te bond, indicating that more energy is required to break the Ta-Te bond, resulting in better thermal stability [20,21]. Therefore, the thermal stability of Sb_3_Te_1_ films was improved after doping with Ta elements.

### 3.2. Device Performance

**Figure 6:**
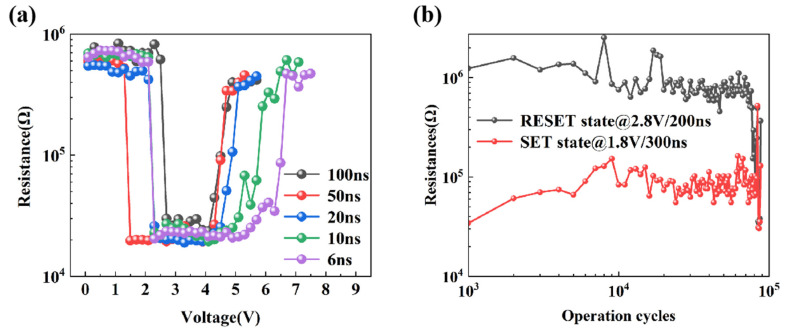
(**a**) The resistance–voltage characteristics of TaST31-2-based T-shaped PCM devices varying pulse widths. (**b**) Durability characteristics of TaST31-2-based PCM T-shaped devices.

The resistance–voltage (R-V) curve for a T-shaped device based on TaST31-2 material is depicted in Figure 6a. The devices based on TaST31-2 material can even perform SET and RESET at ultra-fast pulse widths of 6 ns, allowing for high-speed operation. Therefore, the Ta-doped Sb_3_Te_1_ material’s electrical performance is more dominant than that of GST (10 ns) [22]. It is also evident by seeing the R-V curves that the Ta-doped Sb_3_Te_1_ material requires less voltage to operate in the SET/RESET state. The device performs best at a SET voltage of 2.5 V and a RESET voltage of 4 V for a pulse width of 100 ns. The highest and lowest resistances fall within a window of 1.7 orders of magnitude under these circumstances, which is sufficient for logical differentiation. Figure 6b shows the endurance performance of a T-shaped device based on TaST31-2 material. Set a 300 ns/1.8 V SET pulse and a 200 ns/2.8 V RESET pulse as the device’s operating conditions. The device can be tested to hold for 8 × 10^4^ cycles before a failure occurs. Consequently, the following conclusions are summarised from the above experiments, the switching speeds and SET/RESET voltages always have a huge advantage over GST materials in TaST31-2 device tests.

## 4. Conclusions

The properties of Ta-doped Sb_3_Te_1_ materials for PCM applications are examined in this paper. The crystallization temperature increases with the amount of Ta doping, indicating that the element Ta improves thermal stability. The XRD results can be analyzed to show that Ta doping does not change the crystal structure of Sb_3_Te_1_ material and results in the refinement of the grains. Transmission electron microscopy images show more visually that the size of the crystal grains has become smaller in the TaST31 material, verifying the XRD test results. This reduction in grain size contributes to lower thermal conductivity and lower resistance drift, thus improving the heating efficiency and reliability of the device. X-ray reflectivity (XRR) experiments showed a thickness change (1.01%) and the density change (3.39%) for the TaST31-2 films, which is advantageous to the endurance of the material. XPS experiments illustrate that more Ta-Te bonds with higher bonding energy, verifying the conclusion that the doping of Ta enhances the thermal stability of the material. The PCM device based on TaST31-2 achieves 6 ns operation speed and 8 × 10^4^ cycles. In summary, the TaSb_3_Te_1_ material holds great promise for phase change memory applications.

## Data Availability

Not applicable.
